# Performance, Agro-Morphological, and Quality Traits of Durum Wheat (*Triticum turgidum* L. ssp. *durum* Desf.) Germplasm: A Case Study in Jemâa Shaïm, Morocco

**DOI:** 10.3390/plants14101508

**Published:** 2025-05-17

**Authors:** Khadija Manhou, Mona Taghouti, Rachid Moussadek, Houda Elyacoubi, Sahar Bennani, Abdelmjid Zouahri, Ahmed Ghanimi, Hatim Sanad, Majda Oueld Lhaj, Driss Hmouni, Houria Dakak

**Affiliations:** 1 Laboratory of Natural Resources and Sustainable Development, Department of Biology, Faculty of Sciences, Ibn Tofail University, Kenitra 14000, Morocco; elyacoubihouda@gmail.com (H.E.); hmouni.driss@uit.ac.ma (D.H.); 2 Research Unit on Environment and Conservation of Natural Resources, Regional Center of Rabat, National Institute of Agricultural Research, AV. Ennasr, Rabat 10101, Morocco; r.moussadek@cgiar.org (R.M.); abdelmjid.zouahri@inra.ma (A.Z.); houria.dakak@inra.ma (H.D.); 3 Laboratory of Genetic Improvement and Wheat Quality, Agronomic Research Center of Rabat (INRA), AV. Ennasr, Rabat 10101, Morocco; mouna.taghouti@inra.ma (M.T.); saharbennani@gmail.com (S.B.); 4 International Center for Agricultural Research in the Dry Areas (ICARDA), Rabat 10100, Morocco; 5 Laboratory of Materials, Nanotechnologies and Environment, Department of Chemistry, Faculty of Sciences, Mohammed V University in Rabat, Rabat 10101, Morocco; a.ghanimi@um5r.ac.ma; 6 Laboratory of Process Engineering and Environment, Faculty of Science and Technology Mohammedia, University Hassan II of Casablanca, Mohammedia 28806, Morocco; hatim.sanad99@gmail.com (H.S.); majdaoueldlhaj1999@gmail.com (M.O.L.)

**Keywords:** durum wheat, inter-group genetic diversity, landraces, elite lines, international lines, agro-morphological traits, protein content, gluten content and grain yield

## Abstract

The productivity and resilience of durum wheat have been enhanced through the selection of accessions, optimizing agronomic and quality traits to address environmental challenges. This study evaluates the performance of 219 durum wheat accessions, including 120 elite lines from a national breeding program (G1 to G120), 63 international lines (G121 to G183), 27 Moroccan varieties (including Faraj, Karim, Tomouh, Marzak, Amria, Chaoui, IRDEN, and others), and nine landraces (G211 to G219, from Imilchil, Rich, and Taounate regions). Trials were conducted at the Jemâa Shaïm experimental station (INRA-Morocco) with an “Alpha lattice” design and two replications. Significant correlations were observed between spike length (SL) and number of spikelets per spike (SPS) (*r* = 0.950; *p* < 0.001), and between grain yield (GY) and thousand-kernel weight (TKW) (*r* = 0.530; *p* < 0.01), while no correlation was found between quality parameters and GY (*r* = 0.010; *p* > 0.05). Principal component analysis (PCA) revealed that agronomic traits explained 77.12% of variability, while quality traits accounted for 95.54%. Elite lines exhibited a high yellow pigment index (14.90), important for technological quality. Traditional landraces performed well in spike length (8.78 cm), thousand-kernel weight (50.23 g), protein content (17.07%), and gluten content (36.90%). Moroccan varieties such as Faraj achieved a grain yield of 6.12 t/ha, while international lines showed the highest SDS value (9.39 mL). These findings highlight the potential of diverse accessions for developing high-yielding, high-quality durum wheat.

## 1. Introduction

Durum wheat (*Triticum turgidum* L. ssp. *durum* Desf.) is a vital crop of significant economic and agricultural importance, particularly in regions with limited water availability [1,2]. Its genetic diversity plays a crucial role in improving performance under diverse environmental conditions. Recent studies on durum wheat have identified several agro-morphological traits—such as plant height, heading date, and kernel weight—as key indicators of yield potential and grain quality [3,4]. Furthermore, genetic diversity has been explored in relation to qualitative traits, including grain protein content and dough properties, both of which are essential for evaluating grain quality [5]. The application of quantitative trait loci (QTL) mapping and molecular breeding tools, such as marker-assisted selection (MAS), has significantly enhanced the identification and utilization of these important traits [6,7]. These advancements have greatly contributed to the development of durum wheat varieties with improved adaptability to varying growing conditions, resulting in better yields and higher grain quality [8].

Despite these advancements, durum wheat production faces several challenges, particularly in semi-arid regions, where abiotic stresses such as drought and salinity severely impact both yield and grain quality [9]. Durum wheat (*Triticum durum* Desf., 2n = 4x = 28, AABB), the cultivated form of *T. turgidum*, is an allotetraploid species that originated approximately five hundred thousand years ago [10,11,12]. Over time, its adaptation to diverse environmental conditions has cemented its role as a key cereal crop, especially in Mediterranean regions. Breeding programs have primarily focused on improving tolerance to abiotic stresses, while also enhancing grain yield and quality. In this context, durum wheat plays a pivotal role as the main cereal crop cultivated in Mediterranean regions [13,14]. Although its global production constitutes less than 5% of total wheat production, it holds considerable socio-economic importance in regions such as North Africa, the Middle East, and the Mediterranean basin, where it serves as both a crucial source of income for farmers and a staple food for millions of people [15,16]. In Morocco, durum wheat is especially significant due to its relative adaptability to harsh climatic conditions and its high economic value. However, the increasing threat of salinity and water scarcity, exacerbated by climate change, poses a growing challenge to the sustainability of durum wheat production, emphasizing the urgent need for resilient varieties and more effective breeding strategies [17,18,19,20].

The selection of high-yielding durum wheat germplasm should not be based solely on grain yield [21,22]. Agro-morphological parameters and quality traits, such as awn length, spike length, grain weight, nutritional quality, and resistance to abiotic stresses, are essential for identifying and developing high-performing varieties, particularly in environments subject to climatic variability and abiotic constraints. These traits are often more heritable and stable than yield itself, facilitating more effective indirect selection and helping to mitigate challenges related to the polygenic complexity of grain yield and genotype × environment interactions [23,24]. Consequently, modern breeding programs aim not only to optimize yield potential but also to enhance resilience and grain quality, particularly in regions affected by salinity and drought. By integrating these traits into breeding efforts, a holistic approach is adopted that optimizes both agronomic and qualitative performance, while improving adaptability to specific agricultural systems [25].

The genetic diversity of durum wheat is a crucial resource for improving its resilience to abiotic stresses. Local varieties and traditional populations, often underutilized, represent valuable sources of genes that confer enhanced tolerance to salinity and drought. Furthermore, advancements in marker-assisted selection (MAS) and genomics now allow for the identification of high-performing genotypes, speeding up the development of cultivars adapted to semi-arid conditions. These technologies also facilitate the selection of important agro-morphological and quality traits, such as protein content and gluten quality, which are essential for the food industry [26,27].

International collaborations, particularly with CIMMYT (International Maize and Wheat Improvement Center) and ICARDA (International Center for Agricultural Research in the Dry Areas), have been instrumental in introducing improved durum wheat lines and providing access to diverse germplasm, which combine resilience and high yield under abiotic stress conditions [28,29,30]. These strategic partnerships have enabled the selection of durum wheat varieties specifically adapted to the local and regional conditions of Morocco, offering viable solutions to enhance sustainable agricultural production in semi-arid zones. Several Moroccan durum wheat varieties have been registered in the national catalog, ensuring their availability to local farmers and fostering ongoing improvements in yield within the country. These varieties are chosen not only for their adaptability to Morocco’s unique environmental conditions but also for their ability to maintain high grain quality, which is essential for the semolina and pasta industries [30,31]. The breeding initiatives led by ICARDA focused on developing genotypes that could withstand environmental challenges, particularly by optimizing both grain and biomass yields, while ensuring stability under saline stress conditions. These programs integrated local varieties and derived pure lines to transfer salt tolerance traits into an already well-adapted germplasm. Characterization of landraces involved isolating individual lines from locally grown mixtures. Seeds from the most productive lines were then multiplied and disseminated as new varieties, improving agronomic traits and resilience in challenging environments [32,33,34].

Despite significant breeding efforts, the genetic diversity of durum wheat remains insufficiently explored in some regions, limiting the ability to identify germplasm that is better adapted to harsh environmental conditions, particularly salinity and drought. A deeper understanding of this diversity is essential for enhancing breeding programs, enabling them to more effectively address the growing challenges of food security and agricultural sustainability. This research focuses specifically on the challenges posed by salinity and water scarcity in semi-arid regions, aiming to develop durum wheat varieties that are resilient to abiotic stresses. By examining the inter-class variability of agro-morphological and quality traits, this study contributes to the creation of high-performing germplasm. Additionally, it analyzes the physico-chemical parameters critical to semolina and pasta quality, which are vital for enhancing the socio-economic value of durum wheat in climate-impacted areas. This comprehensive approach is in line with the increasing need for sustainable agricultural practices and resilient crop varieties to mitigate climate-induced stresses [35].

This study aims to characterize the inter-class variability of agro-morphological and quality traits within a large collection of durum wheat, encompassing elite accessions, international lines, Moroccan varieties, and foreign varieties. Additionally, it focuses on analyzing the performance of physico-chemical parameters that influence semolina and pasta quality. Conducted at the Jemâa Shaim experimental site, the research seeks to identify the highest-performing germplasm in terms of both yield and quality, ultimately contributing to the development of resilient varieties and sustainable agricultural practices in semi-arid regions. The objectives of this study are as follows: (1) analyze the performance of agro-morphological traits across different groups within the durum wheat collection, (2) evaluate the performance of physico-chemical characteristics affecting semolina and pasta quality among the groups, (3) identify the most promising germplasm based on agro-morphological and quality traits, (4) apply multivariate statistical analyses, including ANOVA, Duncan test, correlation, and cluster analysis based on PCA, to distinguish and classify the top-performing groups for each parameter, and (5) provide recommendations to promote the adoption of high-performing germplasm and sustainable agricultural practices to enhance durum wheat productivity in semi-arid regions.

## 2. Results

### 2.1. Descriptive Statistics of Agro-Morphological and Quality Traits Measured Across All Wheat Lines

**Figure 1:**
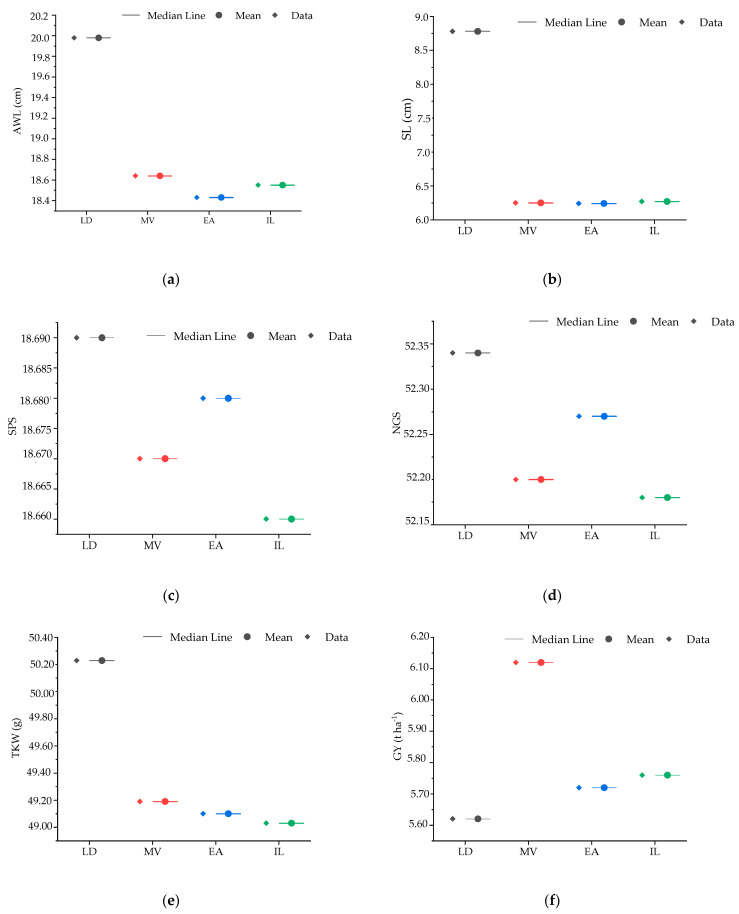
Interval plot (mean with 95% confidence intervals) of agro-morphological parameters for 219 accessions and the four studied lines (box on the right, data on the left). (**a**) Awn length; (**b**) spike length; (**c**) number of spikelets per spike; (**d**) number of grains per spike; (**e**) thousand-kernel weight; and (**f**) grain yield; LD: landraces, MV: Moroccan varieties, EA: elite accessions, and IL: international lines.

**Figure 2:**
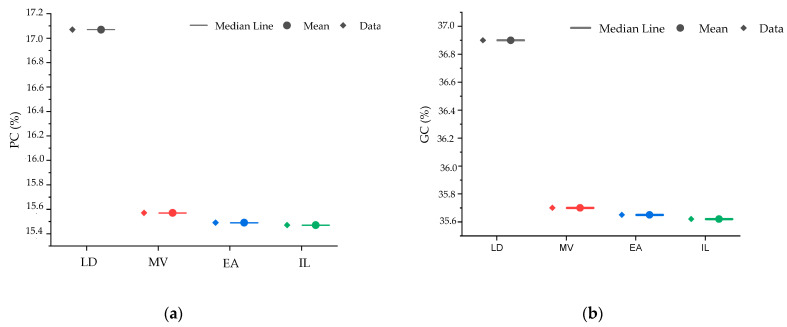

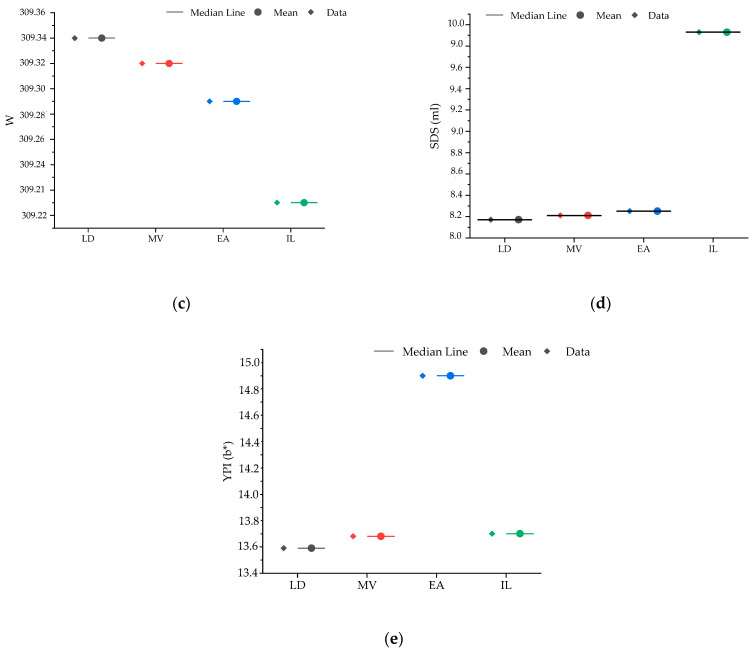
Interval plot (mean with 95% confidence intervals) of quality parameters for 219 accessions and the four studied lines (box on the **right**, data on the **left**). (**a**) Protein content; (**b**) gluten content; (**c**) bread-making strength; (**d**) gluten strength; and (**e**) yellow pigment index; LD: landraces, MV: Moroccan varieties, EA: elite accessions, and IL: international lines.

The results of the descriptive statistics and ANOVA for the agro-morphological and quality parameters of the different wheat germplasm types, including the mean, minimum and maximum values, coefficient of variation (CV), standard deviation (SD), and significance levels, are summarized in Table 1. The interval plots shown in Figure 1 illustrate these variations for agro-morphological parameters, while Figure 2 presents the differences in quality traits.

For awn length (AWL), the mean is 17.22 cm, ranging from 11.34 cm to 23.10 cm, with a coefficient of variation (CV) of 6.6%. ANOVA revealed a highly significant difference (*p* < 0.01), with landraces exhibiting the highest value (19.98 cm), significantly surpassing all other germplasms, while the other groups belonged to the same statistical category. Spike length (SL) averages 8.45 cm, with values from 4.85 cm to 12.05 cm, and a CV of 12.1%. A significant difference was observed (*p* < 0.01), with landraces showing the highest value (8.78 cm), while the other groups did not differ significantly. The number of spikelets per spike (SPS) has an average of 18.88, ranging from 12.05 to 25.70, with a CV of 6.6%. ANOVA did not show a significant difference (*p* > 0.05), indicating homogeneity among the germplasm groups. Thousand-kernel weight (TKW) varies between 23.70 g and 30.65 g, with an average of 27.18 g and a CV of 12%. A highly significant difference (*p* < 0.001) was detected, with landraces having the highest value (50.23 g), while the remaining groups displayed comparable performances. The number of grains per spike (GNS) has a mean of 24.64, ranging from 19.02 to 30.25, with a CV of 4.80%. No significant differences were noted among the groups (*p* > 0.05). Grain yield (GY) averages 5.37 t ha^ࢤ1^, ranging from 1.38 t ha^ࢤ1^ to 9.35 t ha^ࢤ1^, with a CV of 19.70%. A significant difference (*p* < 0.001) was observed, with Moroccan varieties being the most productive, reaching 6.12 t ha^ࢤ1^, while no significant differences were found among the other germplasm types.

Regarding quality parameters, gluten content (GC) has a mean of 37.08%, with values ranging from 26.05% to 48.10%, and a CV of 10.40%. ANOVA showed a significant difference (*p* < 0.01), with landraces having the highest gluten content (36.90%), surpassing the other groups, which were grouped together. Protein content (PC) averages 15.53%, with values between 12.10% and 18.95%, and a CV of 7.40%. A significant difference (*p* < 0.01) was observed, with landraces exhibiting the highest protein content (17.07%), significantly surpassing all other germplasms. The other groups, including Moroccan varieties, international lines, and elite accessions, were classified together. Bread-making strength (W) averages 297.70 mL, with values ranging from 184.20 mL to 411.20 mL, and a CV of 13.20%. ANOVA indicated no significant differences (*p* > 0.05), confirming homogeneity across all groups. Gluten strength (SDS) has a mean of 8.55 mL, with values ranging from 4.55 mL to 12.55 mL, for a CV of 6.80%. A significant difference (*p* < 0.05) was observed, with international lines exhibiting the highest SDS value (9.39 mL). Finally, the yellow pigment index (YPI) averages 13.93, with values ranging from 11.06 to 16.80, and a CV of 8.50%. ANOVA showed significant differences (*p* < 0.01), with elite accessions having the highest yellow pigment index (14.90), significantly exceeding the other germplasms.

#### Correlation Analysis of Agro-Morphological and Quality Parameters Across All Lines

A Pearson correlation matrix was constructed using the actual values of the selected agro-morphological and technological quality parameters for statistical analysis, as shown in Table 2. These parameters include awn length (AWL), spike length (SL), number of spikelets per spike (SPS), grain number per spike (GNS), thousand-kernel weight (TKW), grain yield (GY), protein content (PC), gluten content (GC), bread-making strength (W), gluten strength (SDS), and the yellow pigment index (YPI).

A very highly significant positive correlation was observed of GY with spike length (SL) (*r* = 0.769; *p* < 0.001). GY also showed a highly significant positive correlation with awn length (AWL) (*r* = 0.526; *p* < 0.01). Another important result concerns the highly significant positive correlation between GY and the number of glumes per spike (SPS) (*r* = 0.646; *p* < 0.001). A significant positive correlation was found between GY and TKW (*r* = 0.530; *p* < 0.01). However, no significant correlation was observed between GY and GNS (*r* = 0.155; *p* > 0.05). The data regarding SL measurements showed a very highly significant positive correlation with SPS (*r* = 0.950; *p* < 0.001). Similarly, the data regarding AWL showed a significantly positive correlation with SPS (*r* = 0.414; *p* < 0.01). The data regarding the grain number per spike (GNS) showed no significant correlation with SPS (*r* = 0.161; *p* > 0.05) or with SL (*r* = 0.166; *p* > 0.05). Worthy of note is the significantly negative correlation between TKW and GNS (*r* = −0.498; *p* < 0.01), further reinforced by the strong negative correlation between GY and GNS (*r* = −0.791; *p* < 0.001).

From a technological quality perspective, the correlation analysis between the various parameters revealed several noteworthy relationships (Table 2). Gluten content (GC) displayed a highly significant positive correlation with protein content (PC), with a correlation coefficient of (*r* = 0.863; *p* < 0.001). The latter also showed a significant positive correlation with bread-making strength (W), as indicated by (*r* = 0.410; *p* < 0.01). Similarly, PC demonstrated a significant positive association with W, with (*r* = 0.438; *p* < 0.01). A significant positive correlation was observed between PC and TKW (*r* = 0.420; *p* < 0.01). However, no significant correlation was observed between protein content (PC) and gluten strength (SDS) (*r* = 0.125; *p* > 0.05), nor between protein content (PC) and the yellow pigment index (YPI) (*r* = 0.143; *p* > 0.05). Similarly, no relationship was found between SDS and W (*r* = −0.112; *p* > 0.05) or between YI and W (*r* = −0.079; *p* > 0.05). A significant positive correlation was found between GY and YI (*r* = 0.430; *p* < 0.01). Finally, the analysis revealed highly significant positive correlations between GC and YPI (*r* = 0.825; *p* < 0.001), as well as between SDS and YI (*r* = 0.831; *p* < 0.001).

### 2.2. Principal Component Analysis

**Figure 3:**
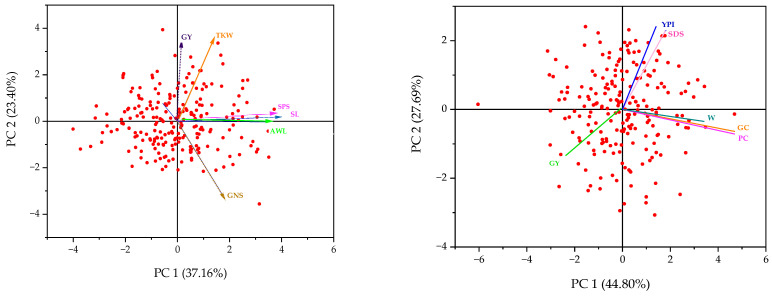
Principal component analysis of agro-morphological (**left**) and quality (**right**) parameters. AWL: awn length; SL: spike length; SPS: number of spikelets per spike; GNS: number of grains per spike; TKW: thousand-kernel weight; GY: grain yield; PC: protein content; GC: gluten content; W: bread-making strength; SDS: gluten strength; YPI: yellow pigment index. Each axis (ACP) represents agro-morphological parameters and quality parameters, with grain yield included for both.

Principal Component Analysis (PCA) was conducted to examine the dispersion of the 225 genotypes in the collection and to identify the agro-morphological and quality traits contributing to their structuring (Figure 3). The objective was to explore genetic variability within the different categories of genetic resources and to determine the traits that contribute the most to grain yield. The first three principal components explained 37.16%, 23.40%, and 16.56% of the total variance, respectively, cumulatively accounting for 77.12% of the total variance of agro-morphological parameters. The PCA revealed correlations between these traits, providing a better understanding of their contribution to genetic diversity. In particular, the vectors representing awn length (AWL) and spike length (SL) were very close, indicating a strong correlation between these two parameters (angle < 90°). Moreover, a significant positive correlation was observed between thousand-kernel weight (TKW) and grain yield (GY), highlighting the key role of TKW in contributing to yield. Regarding quality parameters, the first principal component (PC1) explained 44.80% of the variance, followed by PC2 (27.69%) and PC3 (23.05%), cumulatively accounting for 95.54% of the total variance for quality traits. A strong positive correlation was observed between protein content (PC), gluten strength (SDS), and bread-making strength (W), with the vectors associated with these traits being very close. However, none of these quality parameters directly contribute to grain yield.

### 2.3. Genotypic Classification According to Agro-Morphological and Quality Performances

**Figure 4:**
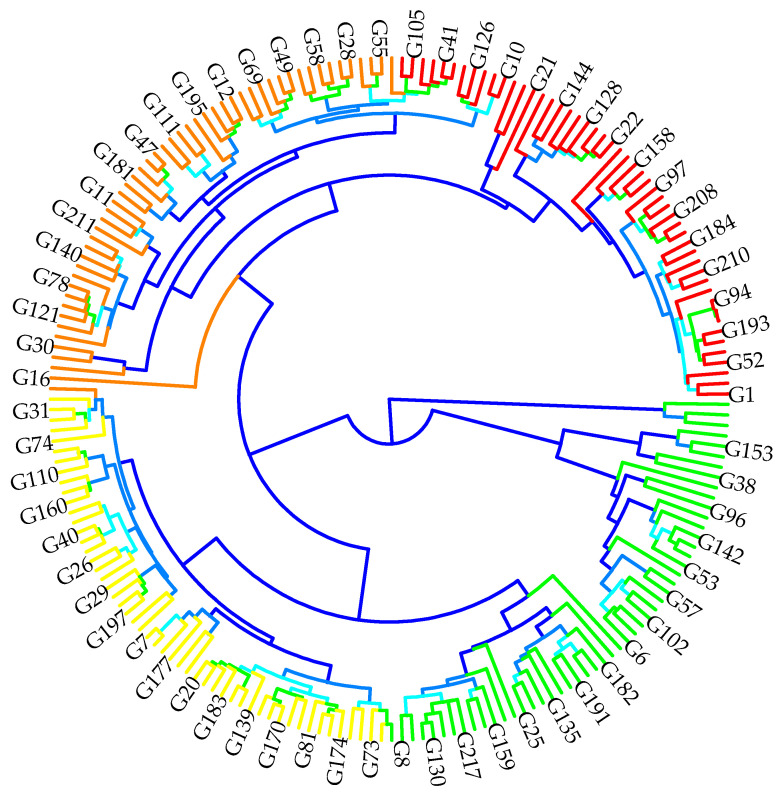
Circular hierarchical cluster analysis (HCA) dendrogram grouping genotypes based on agro-morphological and quality parameters: each color represents a distinct group of genotypes with similar characteristics. G1–G120 representing elite accessions; G121–G129 representing landraces; G130–G156 representing Moroccan varieties; G157–G219 representing international lines.

The hierarchical cluster analysis (HCA) classified the 219 durum wheat accessions into distinct groups based on their agro-morphological and quality traits (Figure 4). The first group included nine landrace genotypes, which had the highest awn length (AWL) and spike length (SL) but exhibited the lowest thousand-kernel weight (TKW) and grain yield (GY). The second group included 27 Moroccan varieties, which had high grain yield (GY), followed by international lines, but showed the lowest awn length (AWL). The third group included the 120 genotypes constituting the elite accessions, which had the highest yellow pigment index (YI) but exhibited the lowest spike length (SL). The fourth group included 63 international lines, which had high gluten strength (SDS) and bread-making strength (W) but displayed the lowest thousand-kernel weight (TKW). Regarding protein content (PC) and gluten content (GC), the landrace genotypes showed the highest values, whereas these parameters were lower in the other groups.

**Figure 5:**
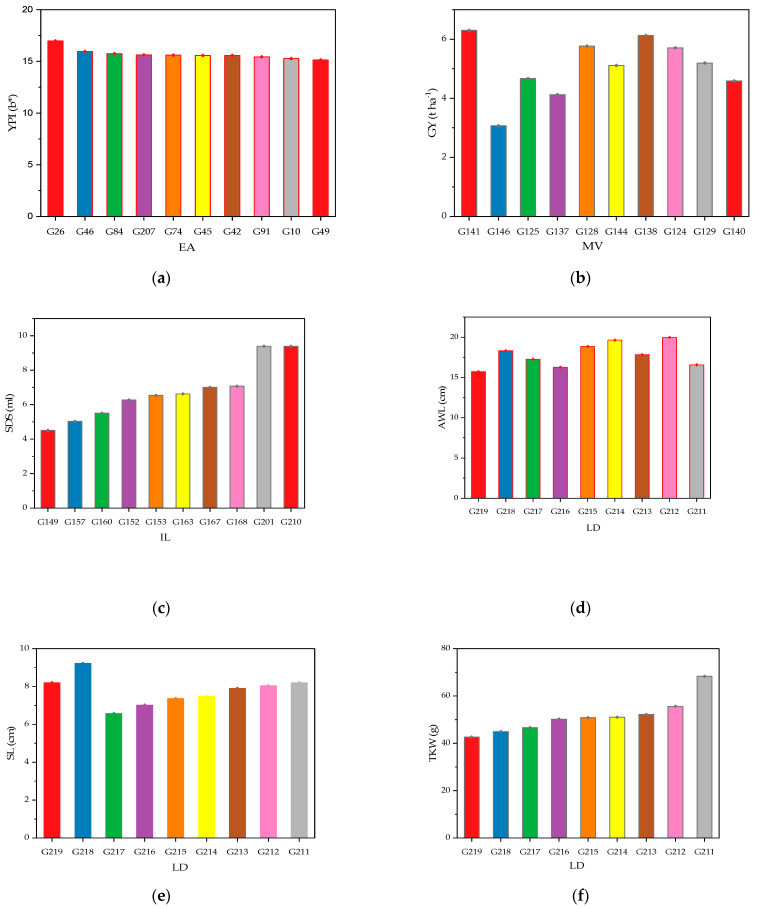

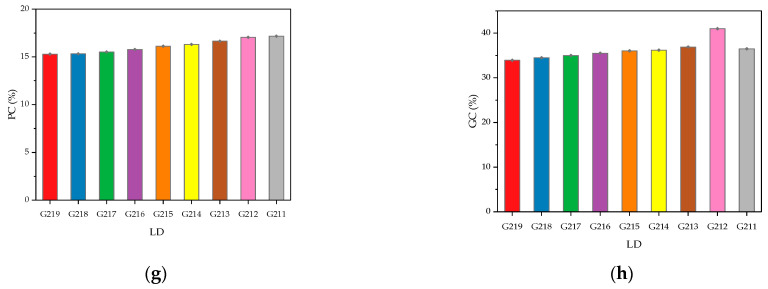
Grouped mean with standard error illustrating the performance variability of genotypes across durum wheat groups. (**a**) Yellow pigment index; (**b**) grain yield; (**c**) gluten strength; (**d**) awn length; (**e**) spike length; (**f**) thousand-kernel weight; (**g**) protein content; (**h**) gluten content; LD: landraces, MV: Moroccan varieties, EA: elite accessions, and IL: international lines.

Thus, Figure 5 illustrates the genotypes constituting each group, where 10 genotypes were selected through screening for each group, including the best- and lowest-performing ones for each parameter, along with other genotypes showing intermediate values. For each group, the best-performing genotype for each parameter is presented based on a thorough analysis of the genotypes constituting the group. This analysis helps to identify the most promising accessions based on their agro-morphological and qualitative characteristics, while emphasizing the variability within each group. The elite accessions (G1 to G120) stood out for their performance in terms of the yellow pigment index (YPI), with genotype G26 exhibiting the highest value, while G49 recorded the lowest. In terms of grain yield, the Moroccan varieties (G121 to G147) demonstrated the highest yield, along with genotype G141 (corresponding to the Faraj variety), while G146 (Jawhar variety) showed the lowest yield. The landraces (G211 to G219) distinguished themselves with superior morphological traits. They exhibited the best awn length (AWL), with G212 presenting the highest value, while G219 had the shortest awn length. In terms of spike length (SL), G218 showed the greatest length, while G217 had the smallest. Regarding thousand-kernel weight (TKW), the landraces recorded the best values, with G211 showing the highest value and G219 the lowest for this parameter. Furthermore, these genotypes exhibited the highest protein content and gluten levels, with G211 and G212 showing the best values for both parameters. Finally, the international lines (G148 to G210) were distinguished by a high gluten content and strong bread-making quality but showed relatively low values in terms of thousand-kernel weight. However, these lines did not display extreme values in other parameters compared to the previous groups.

## 3. Materials and Methods

### 3.1. Experimental Site, Experimental Design, and Management Practices

The experiment was conducted at the Jemâa Shaim experimental site, which is part of the Regional Center for Agronomic Research and located 40 km from Safi, Morocco. This site, covering 320 hectares at an elevation of 168 m (32°40′ N, 10°00′ W), was selected due to its representative agroecological conditions, especially its arid climate and soil characteristics, which are suitable for agricultural trials. The region falls within a dryland agroecological zone, characterized by a very short growing season, droughts, and extreme temperatures, in addition to challenges such as leaf rust and Hessian fly infestations. Climate data from 2015 to 2019 show temperatures ranging from a minimum of 8 °C to a maximum of 35 °C, with an average annual rainfall of 256 mm. The 2017–2018 growing season, which was particularly favorable, benefited from significant rainfall for crop growth. The soils, primarily calcareous cambisols, are typical of dryland agricultural systems, making this site highly relevant for agronomic studies. Agronomic management practices included soil preparation, fertilization, and weeding. Fertilization involved applying a 10-28-10 (N-P-K) complex fertilizer at a rate of 150 kg/ha, followed by ammonium nitrate (33.5% N) at a rate of 20 kg/ha. Weeding was done manually. The previous crop was fallow. Sowing was done conventionally, at a density of 250 seeds/m^2^, under rainfed conditions, in accordance with the site’s agro-climatic characteristics. The experiment followed an Alpha lattice design with two replications. Each replication consisted of 219 elementary plots, with each plot corresponding to an accession sown in two 2 m-long rows spaced 30 cm apart, covering an area of 1.2 m^2^. A 1 m spacing was maintained between plots, and a 2 m distance was kept between blocks to ensure uniform experimental conditions. Preventive fungicide applications were also made to control disease incidence, particularly leaf rust.

To provide a clearer characterization of the climatic conditions during the experiment, Table 3 presents the recorded meteorological data from the Jemhâa Shaim station for the 2017–2018 growing season, including monthly variations in rainfall and temperature. Total precipitation during this period was 232.4 mm, with the highest rainfall recorded in February (62.8 mm) and the lowest in June (0 mm), indicating a notable reduction in water availability during the spring months. Temperature fluctuations ranged from a minimum of −0.1 °C in January to a maximum of 36.5 °C in April, illustrating the exposure to cold spells during winter and extreme heat in the spring. The critical period for crop growth occurred in April and May, characterized by high temperatures (with a peak of 36.5 °C in April and 29.4 °C in May) and reduced rainfall—factors which could negatively impact yield and grain filling. Throughout the experiment, the growth stages of the durum wheat were monitored using the BBCH scale for cereals, enabling precise classification of the plant’s phenological phases [36]. This classification was essential for determining the optimal timing for nitrogen (N) application and harvesting.

### 3.2. Plant Material

The plant material used in this study consists of a set of 219 durum wheat (*Triticum turgidum* L. var. *durum*) accessions, selected based on their adaptability to local agro-ecological conditions and their potential for breeding programs. This collection is divided into four distinct groups, ensuring a diverse representation of geographic and genetic origins. The genotypes were classified as follows: 120 elite accessions, including a mixture of Moroccan breeding lines and international lines. Among the international lines, 28 genotypes (G148 to G175) originated from the International Durum Screening Nursery (IDSN), while 35 genotypes (G176 to G210) were obtained from the International Durum Yield Nursery (IDSYN); 23 Moroccan varieties, developed through the national breeding program; four foreign varieties, registered in the Moroccan national catalogue, obtained from institutions other than INRA but approved by the national registration system; and nine landraces (G211 to G219), with G211 to G217 originating from Imilchil, G218 from Rich, and G219 from Taounate. The genetic origins, pedigrees, and key agronomic traits of the durum wheat varieties used in this study are summarized in Table 4.

### 3.3. Agro-Morphological Characterization

The agro-morphological descriptors of five types of durum wheat germplasm were evaluated according to the international standards defined by “Bioversity” [37]. The parameters assessed included awn length (AWL), spike length (SL), number of spikelets per spike (SPS), number of grains per spike (GNS), and thousand-kernel weight (TKW). For awn length and spike length, three representative spikes were collected from each plot. The spikes were measured using a graduated ruler in centimeters, with awn length recorded from the insertion point at the base of the spike to its longest tip, and spike length measured from the base to the top. The number of spikelets per spike was manually counted. The spikes were gently rubbed by hand to separate the grains without damaging them, ensuring maximum accuracy in the counting. The number of grains per spike was determined using a Choppin Numigral counter (Choppin, Paris, France), which allows rapid and precise counting of grains without any omission. After counting, the grain weight was measured using a precision balance (Ohaus precision balance). This weight was then used to calculate the thousand-kernel weight (TKW) using the following formula:

(1) $$TKW=\frac{Total\;grain\;weight\times 1000}{Number\;of\;grains}$$

This formula allows the determination of the average thousand-kernel weight from the total grain weight and the number of grains per spike. Finally, after harvest, the total grain weight of each elementary plot was measured. The grain yield per hectare was calculated, which allows the determination of the overall yield based on the cultivated area, essential for evaluating the performance of each genotype under specific growing conditions. The formula used for the calculation is as follows:

(2) $$\mathrm{Grain}\;\mathrm{yield}\;({t\;\mathrm{ha}}^{-1})=\frac{\left(\mathrm{Grain}\;\mathrm{weight}\;\mathrm{per}\;\mathrm{plot}\;(\mathrm{kg})\right)}{\mathrm{Plot}\;\mathrm{area}\;(1.2\;m^{2})}\;\times \;100$$

### 3.4. Quality Parameters Characterization

#### 3.4.1. Whole Grain-Based Parameters

The quality parameters of whole grains, including moisture content (MC), grain gluten content (GGC), grain protein content (GPC), and bread-making strength (W), were determined using the Chopin spectrophotometer (Chopin Technologies, Villeneuve-la-Garenne, France), Infraneo model (Chopin Technologies, Villeneuve-la-Garenne, France), based on near-infrared spectroscopy (NIRS), as described by AOAC [38]. Cleaned and impurity-free whole grains were directly placed into the device’s measurement chamber, ensuring precise and non-destructive analysis of these essential traits. The spectrophotometer analyzes the grains by projecting a beam of infrared light onto their surface and measuring the reflected light. This method relies on the reflectance of radiation emitted at specific wavelengths in the visible or infrared spectrum. The chemical bonds in the material (O-H, N-H, C-H) absorb light at particular wavelengths corresponding to their vibrational frequencies and the transition from the ground state to an excited state. These absorption frequencies collectively form the absorption spectrum. The instrument provides a spectrum within a wavelength range of 750 nm to 1100 nm, with a step size of 2 nm. Results for each parameter are automatically generated and displayed on the device interface. For the measured parameters, the device is pre-calibrated according to international standards: ISO 712:2009 for moisture content (MC), the Kjeldahl method for grain protein content (GPC), and ISO 21415-2:2015 for grain gluten content (GGC) [39,40].

#### 3.4.2. Flour-Based Parameters

The milling was performed using the Cyclone Sample Mill laboratory grinder from UDY Corporation (Fort Collins, CO, USA) (Model: 3010-080P/3010-081P), equipped with two sieves: a 0.5 mm mesh sieve for the yellow index (YI) and moisture analyses, and a 1 mm mesh sieve for the sedimentation test. The yellow pigment index (YI) and gluten strength (SDS) were determined using a physical method based on color analysis of whole durum wheat flour with a colorimeter (CR-S Konica Minolta). The procedure involves filling a Petri dish with semolina as a control sample. Then, a Petri dish is filled with a non-compacted flour sub-sample, which is gently tapped to level the flour and eliminate any air pockets at the bottom of the dish. The dish is placed in the appropriate area of the chromameter, calibrated, and the color indices are measured. The control sample is re-analyzed after every five measurements to ensure result reliability. The results are expressed in terms of luminosity (L*), brown index (a*), and yellow index (b*), according to the standards defined by the International Commission on Illumination (CIE). Color evaluation primarily relies on the measurement of the “L*” and “b*” components, while the brown index (a*) is calculated as 100 − L*, and the yellow index corresponds to b* [41]. Gluten strength was determined by the SDS (Sodium Dodecyl Sulfate) sedimentation test, following the Moroccan standard method (N.M.08.1.217, 1999), which is equivalent to the American Association for Cereal Chemistry (AACC 56–70) method. The principle of this test involves measuring the sedimentation volume formed after a series of stirrings and swelling of proteins, as per the established international standard. For this test, 1 g of durum wheat flour is mixed with 8 mL of Reagent 1 in a 25 mL graduated test tube. The solution is manually stirred for 10 s to ensure homogenization. After starting the stopwatch, the sample is allowed to sediment for 4 min and 30 s, with rapid agitation at 2 min and 30 s. At 4 min and 30 s, a final rapid agitation is performed, followed by the addition of 12 mL of SDS-lactic acid solution. The sample is then placed in a shaker (Vortex Genie 2, Scientific Industries, Bohemia, NY, USA) for further homogenization. Finally, the sample is left to sediment, and the sedimentation volume is measured [42].

#### 3.4.3. Data Analysis

The collected data were subjected to a one-way analysis of variance (ANOVA) to assess significant differences among the four durum wheat genetic resources. The means were compared using Duncan’s test at a 5% significance level with SPSS software (version 2024), which showed significant genetic differences between the groups (inter-class), facilitating the selection of the most performance-efficient genotypes for each parameter. A Pearson correlation matrix was calculated using R to explore linear relationships between the parameters, following the methods outlined in the study “Applying Statistical Methods to Library Data Analysis” [43]. Additionally, advanced multivariate analyses, including principal component analysis (PCA), were conducted using R, following a well-established methodology [44]. Furthermore, a cluster analysis based on PCA was performed to classify the lines into distinct groups according to their agro-morphological and quality traits, aiding in the identification of superior genotypes. These analyses deepened the understanding of interactions between agro-morphological and quality traits, highlighting key parameters influencing yield and grain quality variability.

## 4. Discussion

### 4.1. Variability in Agro-Morphological Parameters and Grain Quality Traits

The findings of this study reveal significant agro-morphological variability and notable differences in quality traits among the evaluated durum wheat genotypes. Key traits such as awn length (AWL), spike length (SL), the number of spikelets per spike (SPS) and grains per spike (GNS), thousand-grain weight (TGW), and grain yield (GY) exhibited considerable genetic diversity. This variability extends to essential quality attributes, including gluten content (GC), protein content (PC), dough strength (W), sedimentation capacity (SDS), and yellow index (YI), which are crucial for food processing. Local landraces demonstrated outstanding performance, particularly in SL, TGW, PC, and GC. These traits are often linked to better adaptation to local environmental conditions, underscoring the value of traditional landraces as vital genetic resources for breeding programs targeting arid regions.

The findings of this study are consistent with those reported in previous research, which demonstrates that landraces exhibit enhanced drought tolerance, allowing for stable yields under conditions of water scarcity [45]. These genetically diverse populations contribute to improved stress resilience and yield stability through the application of modern breeding techniques, including genomic approaches [46]. Having been cultivated for millennia in harsh environments, landraces have preserved genetic traits that bolster their resilience, particularly against drought and heat stress [42]. In contrast to modern varieties, which prioritize high yields and genetic uniformity, landraces offer moderate yet reliable yields, while also playing a significant role in biodiversity conservation and ecosystem sustainability [47]. This observation aligns with the findings of other studies that emphasize the importance of drought tolerance, especially during grain filling, in maintaining productivity under water stress conditions [48]. Such germplasm is essential for breeding programs, providing a valuable resource for developing cultivars suited to extreme environments [49,50]. This view is corroborated by studies such as those conducted by [51,52,53], which found that Ethiopian durum wheat landraces exhibited high genetic diversity and competitive yields, particularly in organic farming systems. Furthermore, agronomic traits like thousand-kernel weight (TKW) play an important, though secondary, role in determining final yield, as highlighted by Zhang et al. [54], who demonstrated that increasing kernel weight was a key factor in improving grain yield in high-yielding winter wheat genotypes.

Thousand-kernel weight (TKW) is a crucial indicator of wheat performance, influencing grain yield, milling quality, seed vigor, and growth, all of which significantly impact overall productivity [55]. Previous studies have highlighted the importance of TKW in determining wheat yield potential, confirming its key role. Alongside TKW, spike length (SL) also contributes significantly to wheat productivity. Longer spikes typically result in higher grain density and a greater number of grains per spike, thus increasing yield. Such findings are supported by research demonstrating that SL enhances wheat’s resilience under water deficit conditions, facilitating better grain adaptation to environmental stresses [56]. Protein content is another critical factor influencing durum wheat quality, particularly for pasta production. Wheat with higher protein content (≥13%) is favored for its superior texture, elasticity, and cooking behavior, which helps maintain firmness and prevents overcooking, mainly due to seed storage proteins such as gliadins and glutenins [57,58]. Other studies have also confirmed that higher protein content improves pasta strength and elasticity, enhancing cooking quality and texture stability [59,60].

Additionally, gluten content, a key protein in wheat, determines flour suitability for both bread and pasta production. The quality and quantity of gluten, as measured by the gluten index, directly influence dough behavior and the texture and stability of the final product, particularly in bread-making, as demonstrated in previous research [61]. The gluten index, which varies among wheat varieties, quantifies both gluten content and its viscoelastic properties, playing a crucial role in determining the quality of biscuits and baked goods [62]. This relationship between dough properties and grain protein levels significantly affects biscuit quality, especially texture and stability during baking, as emphasized by Mamat et al. [63]. In terms of quality traits, Moroccan elite genotypes stand out, particularly regarding the yellow pigmentation index, which is a key factor for the pasta industry. The yellow color, primarily due to carotenoid pigments such as lutein, offers not only visual appeal but also nutritional benefits due to its antioxidant properties [64,65]. A high yellow pigment content is highly desirable for optimal pasta color, which enhances both its commercial appeal and nutritional value [66]. Moreover, glutenin alleles play a critical role in gluten strength, influencing pasta processing quality [67]. Moroccan varieties also demonstrate exceptional resilience, with yields reaching up to 6.11 t/ha, even under water deficit conditions in the arid Jemhâa Shaim region. This highlights their ability to withstand water stress and maintain performance in drought-prone areas. Studies emphasize the importance of selecting drought-tolerant genotypes for stable production in arid regions, underlining the potential of these varieties to ensure food security and support local economies [68,69,70,71]. The adaptability of Moroccan genotypes to extreme environmental conditions further underscores their ability to maintain stable yields in the face of climate change [72,73].

### 4.2. Correlations Between Agro-Morphological Traits in the Context of Different Durum Wheat Genotypes

The results of this study reveal significant correlations between various agronomic traits of durum wheat, which are consistent with previous research on wheat productivity. A positive correlation between thousand-kernel weight (TKW) and grain yield (GY) was observed, aligning with findings from Sen et al. [74] and Shamsi et al. [75], where TKW was identified as a key yield component with a direct impact on grain yield. This suggests that increasing TKW can enhance overall yield, reinforcing its role as a critical determinant of wheat productivity. The correlation between spike length and the number of spikelets per spike (SPS), along with its influence on grain distribution, corresponds with studies on bread wheat, particularly the work of Sourour et al. [71], who emphasized the importance of these traits in improving grain yield. Similarly, the negative correlation between grains per spike (GNS) and thousand-grain weight (TGW) found in this study is consistent with earlier reports, which highlighted a trade-off between grain number and weight, likely due to competition for resources under stressed conditions [72]. However, contrasting results have been observed in other studies, where a positive correlation was found, suggesting that environmental factors play a significant role in shaping this relationship [73]. Regarding quality parameters, our findings on the correlation between protein content (PC) and gluten strength align with studies by Kaur et al. [74], who also reported a strong relationship between gluten quality and yellow pigment content in durum wheat, particularly for pasta production. These results underscore the importance of protein composition, particularly gluten subunits, in determining both dough properties and the visual quality of wheat products. In addition, a positive correlation between protein content and thousand-kernel weight was observed in this study, a relationship previously noted by Bogard et al. [75], highlighting the role of protein in both wheat yield and quality. Finally, the positive correlation between gluten strength and yellow pigment content further supports the findings of Kumar et al. [76], who showed that gluten composition, particularly the balance between glutenin and gliadin proteins, is crucial for both dough quality and product appearance, especially for pasta. This correlation emphasizes the importance of considering both the physical and visual qualities of wheat when selecting high-performance genotypes in breeding programs.

### 4.3. Principal Component Analysis and Hierarchical Classification Analyses According to Agro-Morphological and Quality Performances

The principal component analysis (PCA) and clustering of genotypes based on their agro-morphological and technological traits revealed significant genetic variability among the 225 genotypes studied. This diversity reflects the major contribution of certain traits, which were closely associated with both genetic relationships and phenotypic structuring. The study highlighted correlations between specific morphological traits, suggesting that selection for one trait could influence others, consistent with findings in previous research on Moroccan durum wheat genotypes [77,78,79]. Similarly, a previous study also reported significant phenotypic variability among different bread wheat genotypes, emphasizing the importance of phenological and morphological traits in the differentiation of accessions. This supports the effectiveness of PCA in identifying key traits contributing to the genetic structuring of genotypes, providing a better understanding of their contribution to genetic diversity [80]. Furthermore, the analysis revealed significant relationships between quality traits such as protein content and gluten strength, which were closely linked and emphasized as crucial for wheat quality prediction. These findings are consistent with studies that utilized PCA to distinguish genetic groups in wheat, where protein content and gluten strength were identified as key factors influencing wheat quality [81,82]. This is in line with studies on Ethiopian tetraploid wheat, where these parameters were found to play a significant role in genetic improvement, particularly under varying environmental conditions [83,84,85]. Similarly, other studies on wheat genetic diversity have employed molecular markers to analyze the structuring of accessions, showing that genetic diversity is influenced by the origin of the genotypes and selection pressures. The clusters observed in this study suggest a combined influence of genetic factors and selection on the differentiation of genotypes, reinforcing the findings from previous research on wheat genetic diversity [86,87,88].

### 4.4. Screening and Performance of High-Performing Genotypes in Durum Wheat Lines

Screening genotypes is a crucial step in the genetic improvement of durum wheat, particularly in specific environments such as Jemâa Shaïm, Morocco. Several studies have highlighted the importance of this approach for selecting high-performing genotypes based on both agronomic and quality traits. Previous research has emphasized the essential genetic variability required to select genotypes adapted to local conditions while ensuring optimal grain quality [51,52,53]. These studies have demonstrated the need to identify genotypes that are not only agronomically efficient but also capable of adapting to environmental variations, which is critical for enhancing both yield and grain quality in specific environments. In this study, the landraces (G211 to G219) stood out due to their significant variability in both morphological and quality traits. Notably, genotypes G211 and G212 exhibited exceptional performances in terms of spike length and grain quality, with high protein content and favorable gluten levels, making them particularly valuable for grain quality applications. These genotypes also demonstrated good adaptation to local conditions, with G212 recording the highest spike length and awn length (AWL), a crucial trait for stress resistance. However, variability was also observed in other parameters, such as thousand-kernel weight (TKW), where G211 showed the best performance, while G219 recorded the lowest values. This variability highlights the importance of selecting genotypes not only based on agronomic traits but also on their adaptability to specific environmental conditions. The potential of landraces to improve grain quality while maintaining strong agronomic performance underscores their value as a rich source of genetic variability, essential for breeding programs [89,90,91]. In contrast, Moroccan varieties, particularly the Faraj variety, exhibited high yields, demonstrating strong adaptation to local conditions. This finding is consistent with the results of Manhou et al. [23], who identified that the Faraj variety as capable of maintaining an acceptable yield even under challenging conditions, making it particularly well-suited for moderately saline environments. Elite accession genotypes showed high values for the yellow pigment index (YPI), indicating a greater ability to capture and retain chlorophyll pigments. During genotype screening, G26 displayed the highest value for this index, which is consistent with previous studies linking higher yellow pigment index values to improved photosynthetic efficiency [92,93,94,95]. International lines distinguished themselves through their strong performance in gluten strength, as measured by the sodium dodecyl sulfate (SDS) test, a key parameter for gluten quality. Genotype G201, sourced from the International Durum Screening Nursery (CIMMYT), exhibited the highest values for this criterion, highlighting its potential for producing high-quality gluten. These results align with findings from multiple studies on international lines, which have identified these genotypes as important sources of genetic variability for improving durum wheat quality traits, particularly gluten quality, which is essential for baking products [96,97]. International resources have been widely recognized for their breeding programs focused on improving gluten quality, enabling the development of durum wheat varieties suited to diverse environmental conditions while maintaining consistent grain quality [98,99,100,101].

## 5. Conclusions

This study assessed the genetic and phenotypic diversity of 219 durum wheat accessions, which encompass a wide range of germplasm types, including elite accessions, international lines, Moroccan varieties, and local landraces. The primary objective was to evaluate their performance based on both agro-morphological traits and grain quality. Significant differences were observed among the studied groups, which underscored the considerable genetic variability present in the accessions. This variability presents a valuable resource for future breeding programs, especially in arid and semi-arid environments where agricultural conditions are challenging. Notably, local landraces demonstrated exceptional performance in several key quality parameters, positioning them as promising candidates for developing high-quality durum wheat varieties. These landraces, therefore, represent a crucial genetic pool for the development of varieties that meet both local and international market standards. Among Moroccan varieties, the Faraj variety stood out for its high yield, indicating its strong adaptation to local conditions and its stability under varying environmental stresses. International lines were notably distinguished by their high sedimentation index (SDS), a key indicator of gluten strength, while elite accessions exhibited superior yellow pigment index values, making them suitable for producing wheat with desirable visual traits, especially for semolina or flour production. Statistical analyses confirmed the significant genetic diversity within the studied groups. Principal component analysis (PCA) revealed that agro-morphological traits accounted for a substantial portion of the total variability, while quality traits, such as protein and gluten content, contributed significantly to this differentiation. The results highlight the importance of both genetic and phenotypic diversity in the ongoing improvement of durum wheat. Selecting genotypes that combine favorable agro-morphological traits with high-quality characteristics will be key to developing high-performing varieties that meet the demands of both agricultural and industrial sectors. Additionally, preserving this genetic and phenotypic diversity is crucial for ensuring the sustainability of wheat production, especially in the face of climate change challenges and the growing global demand for food. Future research should build upon these findings by considering inter-annual variations and integrating both phenotypic and genetic approaches. Molecular markers could be employed to gain a deeper understanding of the genetic basis of variability and to refine breeding strategies aimed at developing resilient durum wheat varieties that are specifically tailored to local environmental conditions.

## Figures and Tables

**Table 1:** Variability and ANOVA of agro-morphological and quality traits measured across all studied wheat germplasm.

Parameter	Df	Sum Sq	Mean Sq	*p*-Value	Mean ± SE	Mean	Minimum	Maximum	CV (%)	SD
AWL (cm)	4	5.58	9.93	<0.01	18.90 ^ab^ ± 0.56	17.22	11.34	23.10	6.60	1.16
SL (cm)	4	6.10	1.55	<0.01	6.89 ^ab^ ± 0.22	8.45	4.85	12.05	12.10	3.57
SPS	4	1.92	0.48	>0.05	18.55 ^a^ ± 0.21	18.88	12.05	25.70	6.60	2.18
TKW (g)	4	559.12	136.56	<0.001	49.39 ^abcd^ ± 0.35	27.18	23.70	30.65	12.00	2.77
GNS	4	379.35	97.13	>0.05	52.25 ± 0.07	24.64	19.02	30.25	4.80	1.17
GY (t ha^ࢤ1^)	4	13.67	3.58	<0.001	5.81 ^abcde^ ± 0.11	5.37	1.38	9.35	19.70	2.21
GC (%)	4	64.92	16.23	<0.01	15.90 ^abc^ ± 0.44	37.08	2.05	48.10	10.40	1.76
PC (%)	4	9.56	2.37	<0.01	35.97 ^abc^ ± 0.35	15.53	12.10	18.95	7.40	0.93
W	4	12,686.25	3174.06	>0.05	309.32 ^a^ ± 0.04	297.70	184.20	411.20	13.20	2.17
SDS (mL)	4	21.22	5.59	<0.05	8.51 ^ab^ ± 0.26	8.55	4.55	12.55	6.80	1.16
YPI (b)	4	5.37	1.39	<0.01	13.97 ^ab^ ± 0.33	13.93	11.06	16.80	8.50	0.38

Means (with standard errors, n = 3) and standard deviations sharing the same letter indicate no statistically significant differences at the 0.05 significance level, as determined by comparisons of means using Duncan’s test. Df: degrees of freedom; Sum Sq: sum of squares; Mean Sq: mean square; CV: coefficient of variation; SD: standard deviation; *p*-value associated with the F statistic: not significant: *p* > 0.05, significant: *p* < 0.05, highly significant: *p* < 0.01, and very highly significant: *p* < 0.001.

**Table 2:** Pearson correlations between agro-morphological traits and grain quality in the studied lines.

	AWL	SL	SPS	GNS	TKW	GY	PC	GC	W	SDS	YPI
AWL	1										
SL	0.379 *	1									
SPS	0.414 **	0.950 ***	1								
GNS	0.259 *	0.166	0.161	1							
TKW	0.189	0.195	0.287	−0.498 **	1						
GY	0.526 **	0.769 ***	0.646 ***	−0.791 ***	0.530 **	1					
PC	−0.020	0.030	0.080	0.010	0.420 **	0.020	1				
GC	0.030	0.020	0.060	0.070	−0.010	−0.020	0.863 ***	1			
W	−0.100	−0.110	−0.050	−0.040	0.050	0.020	0.438 **	0.410 **	1		
SDS	−0.060	−0.050	−0.050	−0.050	−0.010	0.010	0.125	0.143	−0.112	1	
YPI	−0.060	−0.060	−0.050	−0.050	−0.010	0.430 **	0.143	0.825 ***	−0.079	0.831 ***	1

AWL: awn length; SL: spike length; SPS: number of spikelets per spike; GNS: number of grains per spike; TKW: thousand-kernel weight; GY: grain yield; PC: protein content; GC: gluten content; W: bread-making strength; SDS: gluten strength, and YPI: yellow pigment index. *p*-value associated with the F statistic: not significant: *p* > 0.05, significant: * *p* < 0.05, highly significant: ** *p* < 0.01, and very highly significant: *** *p* < 0.001.

**Table 3:** Monthly meteorological data recorded at the Jemhâa Shaim station during the 2017–2018 growing season.

Month	Precipitation (mm)	Air Temperature [(°C)]			Growth Stage	BCCH
		Mean	Min	Max		
November 2017	41.20	11.75	3.00	20.50	Sowing	00
December 2017	42.20	12.35	3.30	24.90	Tillering	21
January 2018	46.80	11.98	−0.10	24.50	Tillering	21
February 2018	62.80	11.58	0.60	24.40	1st node ^1^	31
March 2018	52.60	14.75	2.80	26.30	2nd node ^2^	32
April 2018	19.60	16.71	5.40	36.50	Full Flowering	65
May 2018	8.40	18.05	5.50	29.40	Maturity	99
June 2018	0.00	21.04	10.00	31.10	Maturity	99

^1^ First and ^2^ second top-dressing application.

**Table 4:** Genetic origins and characteristics of the durum wheat varieties used in this study.

Code	Wheat Variety	The Official Year of Inscription	Origin/Breeder	Adaptability	Yield Potential (t ha^−1^)	Quality Traits
G121	Anouar	1993	Crossbreeding and selection of Moroccan varieties on introduced material	Large adaptation, irrigated	7.5	PC: 13.5%; Mt: Moderately Resistant BK: Good; SQ: Good
G122	Jawhar	1993	Selection through hybridization with introduced material	Large adaptation, irrigated	7.5	PC: 13.1%; Mt: Moderately Resistant BK: Good; SQ: Good
G123	Amjad	1995	Moroccan crossbreeding with introduced material	Large adaptation, irrigated	7.5	PC: 13.0%; MT: Moderately Resistant BK: Good; SQ: Good
G124	Tarek	1995	Moroccan crossbreeding with introduced material	Large adaptation	7.5	PC: 13.1%; MT: Moderately Resistant BK: Good; SQ: Good
G125	Tomouh	1997	Moroccan selection on introduced material (Oum Rabia 6 from ICARDA)	Arid areas and plateaus	6.0	PC: 14.0%; MT: Moderately Resistant BK: Good; SQ: Good
G126	Karim	1985	Moroccan crossbreeding with CIMMYT material	Large adaptation except in high altitude	7.5	PC: 13.2%; MT: Moderately Resistant BK: Average to Good; SQ: Good
G127	Marzak	1984	Moroccan crossbreeding with CIMMYT material	Large adaptation except in high altitude	7.5	PC: 13.2%; MT: Moderately Resistant BK: Average to Good; SQ: Average
G128	Amria	2003	Moroccan crossbreeding and selection	Semi-arid areas	5.0	PC: 13.4%; MT: Moderately Resistant BK: Good; SQ: Good
G129	Chaoui	2003	Moroccan crossbreeding and selection	Semi-arid areas	6.0	PC: 13.5%; MT: Moderately Resistant BK: Good; SQ: Good
G130	IRDEN	2003	Moroccan crossbreeding and selection	Semi-arid areas	6.0	PC: 13.5%; MT: Moderately Resistant; BK: Good; SQ: Good
G131	Nassira	2003	INRA Morocco	Semi-arid areas	4.5	PC: 15.0%; BK: Good; SQ: Good
G144	Prospero	2007	Florimond Desprez	Favorable areas Semi-arid areas	high	BK: High
G145	Boniduro	2012	Semillas Battle	Favorable areas Semi-arid areas	High	PC: High; Vit.: Superior; GQ: High; YPI: Medium-High
G146	Marjana	1996	INRA selection from CGIAR material	Large adaptation	4.8	PC: 13.2%; MT: Moderately Resistant BK: Good; SQ: Good
G147	Carioca	2005	Serasem-France	Irrigated areas	high	BK: Good
G132	Marouane	2003	Moroccan crossbreeding and selection	Semi-arid areas	6.0	PC: 13.5%; MT: Moderately Resistant BK: Good; SQ: Good
G132	Nassira	2003	INRA Morocco	Semi-arid areas	4.5	PC: 15.0%; BK: Good; SQ: Good
G133	Yasmine	1993	Moroccan crossbreeding on introduced material	Large adaptation	7.5	PC: 13.2%; MT: Moderately Resistant BK: Good; SQ: Good
G134	Ourgh	1995	Moroccan crossbreeding on introduced material	Large adaptation	7.5	PC: 13.5%; MT: Moderately Resistant BK: Good; SQ: Good
G135	Louiza	2011	INRA Morocco	Large adaptation	6.0	PC: 14.8%; BK: Good; SQ: Good
G136	Oued Znati	1949	Selection from a former Moroccan local population	North and mountainous areas	4.0	PC: 14.7%; MT: Moderately Resistant BK: Low; SQ: Medium
G137	Kyperounda	1956	Selection from a former local Moroccan population	Favorable plains, northern regions, and mountainous areas	4.0	PC: 14.0%; Mt: Moderately Resistant BK: Good; SQ: Good
G138	Nachit	2018	INRA Morocco	Favorable areas Semi-arid areas	7.1	PC: 15.0%; BK: Good; SQ: Good
G139	BD 15-13	Seeds of Provence	-	-	-	-
G140	Jabal	Delivered in 2021	JLIBEN CONSULTING	Semi-arid areas	3.0	PC: 13%; BK: Good; SQ: Good
G141	Faraj	2007	INRA Morocco	Favorable areas Semi-arid areas	6.8	PC: 15.3%; BK: Good; SQ: Good
G142	Itri	2016	INRA Morocco	Favorable areas Semi-arid areas	7.0	PC: 14.1%; BK: Good; SQ: Good
G143	Kanakis	2009	Florimond Desprez	Favorable areas Semi-arid areas	High	BK: High

CGIAR: Consultative Group on International Agricultural Research; PC: protein content; Mt: middling resistance; BK: baking quality; SQ: semolina quality; Vit: vitreousness; GQ: gluten quality; YPI: yellow pigment index.

## Data Availability

The data used in this study are available upon request. We encourage interested researchers to contact us for further information.
